# High-Performance Vision Training Improves Batting Statistics for University of Cincinnati Baseball Players

**DOI:** 10.1371/journal.pone.0029109

**Published:** 2012-01-19

**Authors:** Joseph F. Clark, James K. Ellis, Johnny Bench, Jane Khoury, Pat Graman

**Affiliations:** 1 Department of Neurology, University of Cincinnati, Cincinnati, Ohio, United States of America; 2 College of Education, Criminal Justice, and Human Services, University of Cincinnati, Cincinnati, Ohio, United States of America; 3 University Health Services and Department of Athletics, University of Cincinnati, Cincinnati, Ohio, United States of America; 4 Cincinnati Children's Hospital Medical Center, Cincinnati, Ohio, United States of America; 5 Hall of Fame catcher from Cincinnati Reds, Stryker spokesman and http://opdocs.com/OpDocs.com spokesman, Cincinnati, Ohio, United States of America; Universidad Europea de Madrid, Spain

## Abstract

**Purpose:**

Baseball requires an incredible amount of visual acuity and eye-hand coordination, especially for the batters. The learning objective of this work is to observe that traditional vision training as part of injury prevention or conditioning can be added to a team's training schedule to improve some performance parameters such as batting and hitting.

**Methods:**

All players for the 2010 to 2011 season underwent normal preseason physicals and baseline testing that is standard for the University of Cincinnati Athletics Department. Standard vision training exercises were implemented 6 weeks before the start of the season. Results are reported as compared to the 2009 to 2010 season. Pre season conditioning was followed by a maintenance program during the season of vision training.

**Results:**

The University of Cincinnati team batting average increased from 0.251 in 2010 to 0.285 in 2011 and the slugging percentage increased by 0.033. The rest of the Big East's slugging percentage fell over that same time frame 0.082. This produces a difference of 0.115 with 95% confidence interval (0.024, 0.206). As with the batting average, the change for University of Cincinnati is significantly different from the rest of the Big East (p = 0.02). Essentially all batting parameters improved by 10% or more. Similar differences were seen when restricting the analysis to games within the Big East conference.

**Conclusion:**

Vision training can combine traditional and technological methodologies to train the athletes' eyes and improve batting. Vision training as part of conditioning or injury prevention can be applied and may improve batting performance in college baseball players. High performance vision training can be instituted in the pre-season and maintained throughout the season to improve batting parameters.

## Introduction

Baseball is a sport with a tremendous amount of quantitative batting data being generated from batting averages, slugging percentages, and numbers of hits, walks, strike outs and a host of others. In particular, batting is an activity that has rigorous demands for eye-hand coordination requiring concentration and good visual acuity as well as depth perception. There is general agreement that vision training is beneficial to various sports related activities but an objective and quantifiable assessment validating the concept is relatively lacking in the literature [1].

The time it takes for a pitched ball to reach the plate is approximately 0.4 seconds. In that time the batter needs to spot the pitch, assess rotation and direction of the ball to finally make a decision to swing or not [2]. When swinging the bat the batter must consider both the timing of the swing and the angle of the swing.

The swing takes approximately 0.2 seconds [3]. With the velocity of action potentials being approximately 60 m/s and approximately 2 mS needed to cross each synapse, and a minimum of 5 synapses crossed that means it can take as long as 0.03 seconds to process the swing. Therefore, there remains only about 0.17 seconds to decide to swing.

As a physical activity batting is ideal for assessing and quantifying the benefits and affects of high performance vision training [4], [5], [6]. The coaching and vision performance team at the University of Cincinnati decided that enhanced vision performance of the batters would be beneficial to the offensive game of the University of Cincinnati Baseball team and initiated a vision training program in January 2011.

In this paper we report on the methodology used to improve the vision performance of the batters in the University of Cincinnati Baseball team for the 2011 season and compare batting statistics to 2010 team statistics. We found that many batting parameters increased significantly for the team when compared to the opponents' performance as well as the Big East Conference's performance.

## Methods

### Human Subjects

The training of the athletes was performed in accord with standard vision training for all batters as requested by the head coach, Brian Cleary. The task set by the coach was to train and improve batters. No specific defensive training was performed. Also, no consent forms were signed and no controls were used. Comparisons are made from the 2010 team batting statistics to the 2011 team batting statistics; ie before and after vision training for the team. This is an observational study, we make comparisons to the 2010 season because prior to 2011 there was no specialized vision training for the players. The activity has been reviewed by the University of Cincinnati Institutional Review Board and is compliant with all human subjects rules.

Six weeks prior to the season a thrice weekly vision training session was initiated including: Dynavision, Tachistoscope, Brock String, Eyeport, Rotary, Strobe Glasses, Near Far Training, and Saccades.

### Dynavision

The Dynavision is a eye-hand coordination device that tests and improves visual motor skills [7]. We typically perform two one minute sessions on the athletes. The reason for doing multiple sessions is to demonstrate consistency and improvement with the tests. The staged and progressive nature of the tests also helps keep the athletes engaged.

The off the shelf, *A training session is an established Dynavision protocol [7]. It uses traditional eye-hand reaction training to assess visual fields and improve reaction times. This training drill takes one minute. The result is a number of hits in one minute as well as the average reaction time for each hit.

### Tachistoscope

The Tachistoscope is a device that trains the brain to recognize images faster, and loosely correlates to batting average [8]. We flash numbers on a screen, typically starting with 1 number at 0.25 seconds, gradually adding more numbers [up to 4] randomly placed on the screen, and also including different backgrounds at increasingly faster flashes. We also start with simple contrasts such as black on white and make them increasingly more difficult such as darker green letters on a lighter green background. This is called contrast sensitivity and is very effective in training an athlete to recognize objects in his visual field faster.

### Brock String

The Brock string is a classic visual training aid that uses a string and colored balls [5]. Ours was an eight foot string with 5 colored balls. Typically the athlete would have the nearest end of the string by their nose and have it extend away from them parallel to the ground. The athlete needs to focus on the balls, set along the 8” string, back and forth for 1 minute. This requires adaption and convergence of the eyes to find and focus on the balls. The exercise conditions the eye and lens muscles to quickly make adjustments.

### Eyeport

The Eyeport (Exercise Your Eyes, Dove Canyon, CA) is effectively an automated version of the Brock String that has a series of different colored lights [9]. The athlete holds it to his nose and follow the lights to improve his convergence of the eyes and also do lateral and vertical saccades. The device is used as described above for 1 minute intervals, and is basically a stretching and warm-up device for the extra-ocular muscles and for the ancillary muscles which focus the eye.

### Rotary

The rotary (Bernell Corporation of Mishawaka, IN) is a vision pursuit device that has letters and numbers attached by Velcro. It rotates at increasing speed as the athlete improves the ability to call out and point the laser pointer at the appropriate letter/number in alphabetical/numerical order. This is done for 1 minute both clockwise and counterclockwise.

### Strobe Glasses

Strobe glasses are LED lenses that flash and completely block the signal to the eyes as objects are in motion [5]. They are set to flash more rapidly in the initial training stages and are gradually slowed up as the athlete gets adapted to the training. The slower the interval, the more difficult the task due to less visual input due to the LED's interruptions. The brain is forced to visualize where the pitch is going by processing the information it gets from the eyes faster. It is used for batting practice and the effect is that batters report that the pitch seems to be moving slower and they can pick up the ball coming out of the pitchers hand easier.

### Saccades

Saccades are rapid movement of both eyes in the same direction from one object to another voluntarily [5]. We set charts of random letters on a wall, both horizontally and vertically and had the players stand at varying distances and focus from one chart to another, calling out the letters they see in order on a line, alternating from one chart to another for a period of 1 minute. This is done in many forms such as looking over and alternating shoulders after looking forward at another chart. This simulates a fielder chasing a hit ball.

### Near Far Training

Near far training consists of the subject focusing on two different cards approximately 18 inches and 10 feet away [5]. The athletes focus back and forth on the card and count how many iterations they can do. The cards have rows of random letters so that they have to track their progress in a similar fashion to the saccades.

### Preseason Training

Six weeks prior to the start of baseball season all athletes began a thrice weekly 30 minute vision training session. Much of the first week consisted of educating the batters on the methodology for performing the drills safely and effectively. Each athlete was regularly monitored by the Vision Performance Team for effective training habits and to address questions. The sessions typically consisted of 2 Dynavision sessions, 1 Tachistoscope, 2 Brock String, 2 Eyeport, 2 Rotary, 2 Near Far Training, and 2 Saccades. Strobe glasses were not used preseason. Each drill typically lasted 1 minute.

### In Season Training

During the baseball season each athlete engaged in twice weekly sessions of vision training that typically lasted 20 to 30 minutes consisting of approximately 6 to 10 one minute exercise. Training sessions were alternated to include a variation of the exercises described above. The alternation of exercises is a fairly standard philosophy in training to keep the athlete engaged and to avoid boredom from an excessively rigid routine. A typical training session in season would include: 2 minutes on Dynavision, 1 Eyeport session, 1 Tachistoscope, 1 session on Near Far, 1 session on Rotary, 2 sessions on Brock String, and 1 session on Strobe Glasses.

The training pre-season and in season was gently escalated and varied, as the athlete's skill and abilities allowed. It is also a common coaching / training tool to keep an athlete interested and engaged in the training activities. Such variation is justifiable because the goal of the activity was for benefiting the athlete's performance as per coach instructions. An example of an escalation method is to increase the rotational speed of the rotary or the decrease the time of the flash for the Tachistoscope.

### Data Management and Analysis

All data were obtained from publically available sources where college baseball statistics are found. The following URLs are the main sources for data presented and discussed herein:

http://www.gobearcats.com/sports/m-basebl/stats/2010-2011/teamstat.html,

http://www.bigeast.org/fls/19400/stats/baseball/2011/lgsumm.htm,

http://www.bigeast.org/fls/19400/stats/baseball/2010/lgsumm.htm,

http://www.bigeast.org/fls/19400/stats/baseball/2011/lgteams.htm,

http://www.bigeast.org/fls/19400/stats/baseball/2010/lgteams.htm,

http://www.bigeast.org/fls/19400/stats/baseball/2011/lgconf.htm,

http://www.bigeast.org/fls/19400/stats/baseball/2010/lgconf.htm,

Data presented in the tables below and used for analysis were extracted from the above referenced urls.

### Data Analysis

We analyzed two consecutive years the 2010 season and the 2011 season, in order to maximize the number of consistent players in all of the teams. A simple t-test statistic was used to analyze the difference in change for Cincinnati compared to the other Big East teams. For this an underlying normal distribution for the baseball statistics, batting average, slugging percentage and on-base percentage was assumed. All differences are reported as the value for Cincinnati minus the value for their opponents.

## Results

The batting average for the University of Cincinnati baseball team went from 0.251 to 0.285 before and after vision training (Batting average is hits/at bats, excluding walks, fielders choice and sacrifice]. This is a 0.034 improvement in the team's batting average. The rest of the Big East fell 0.034 [Table 1]. This difference is 0.068 with 95% confidence interval [0.021, 0.114], with Cincinnati significantly improving compared to the rest of the conference [p<0.01].

**Table 1:** The data presented represents All University of Cincinnati games and their opponents performance during those games.

	BA	Gp-Gs	A.B.	Run	Hits	2b	3b	H.R.	RBI
2011UC	.285	57	1912	317	545	105	9	35	286
2011Opponents	.280	57	1914	313	536	94	9	24	287
2010UC	.251	58	1905	287	479	89	7	42	251
2011Opponents	.288	58	2020	328	581	104	16	38	294

UC  =  University of Cincinnati. Ave  =  batting average. Gp gs  =  games played games started, A.B.  =  at bat, 2b  =  doubles, 3b  =  triples, H.R.  =  home run, RBI  =  run batted in.

The team batting average went from being ranked 12^th^ in the Big East in 2010 to tied for 4^th^ in 2011. During the same time frame the opponent's batting average slightly decreased from 0.288 in 2010 to 0.280 in 2011. The team rankings in the Big East improved from 7^th^ in 2010 to tied for 4^th^ in 2011 where they had a 0.500 season in 2010 to a 0.526 season in 2011.

The numbers of games played and at bats for the University of Cincinnati was similar between 2010 and 2011 [58 to 57; and 1905 to 1912 respectively]. The number of hits increased 14% whereas the number of hits for the opponents decreased 8%, possibly due to a decrease in at bats of 5% [See Table 1]. Doubles, triples and runs batted in all increased. Home runs decreased for the University of Cincinnati and the opponents [16% and 36% respectively].

In Table 2 we see that the slugging percentage and on base percentage increases from 2010 to 2011. In 2010 the slugging percentage, a reflection of hits with extra bases increased from 0.372 to 0.404, whereas for the opponents it decreased from 0.411 to 0.376. With their opponents slugging percentage falling over that same time frame 0.082 this produces a difference of 0.115 with 95% confidence interval [0.024, 0.206]. As with the batting average, the University of Cincinnati is significantly different from their opponents p = 0.02.

**Table 2:** The data presented represents All University of Cincinnati games and their opponents performance during those games.

	Tot Bse	Slg%	Walk	H.B.P.	S.O.	G.D.P	Ob%	S.F.	S.H	Sb- att
2011UC	773	.404	227	44	382	36	.370	21	33	69–102
2011Opponents	720	.376	208	56	353	38	.364	22	58	45–75
2010UC	708	.372	212	44	418	28	.336	25	28	61–85
2011Opponents	831	.411	174	54	399	16	.355	28	45	59–89

Tot bse  =  total bases, slg%  =  slugging percentage, H.B.P.  =  hit by pitch, S.O.  =  strike out, G.D.P.  =  ground into double play, ob%  =  on base percentage, S.F.  =  sacrifice fly, S.H.  =  sacrifice hit, sb-att  =  stolen base-attempts.

The on base percentage for the University of Cincinnati increased by 0.034 points, while for the rest of the Big East on base percentage fell by 0.034, Table 3. This produced a difference of 0.068 with 95% confidence interval for the on base percentage of [0.024, 0.111] with University of Cincinnati again different from the rest of the Big East p<0.01. Strikeouts for the University of Cincinnati and opponents decreased 8.6% and 11.5% respectively.

**Table 3:** Comparison of 2010 season to 2011 season: Cincinnati [Cin] versus the remainder of the Big East [BE].

	2010		2011		Change		Difference [95% CI]
	Cin	BE	Cin	BE	Cin	BE	
Batting average	0.251	0.305	0.285	0.272	0.034	−0.034	0.068 [0.021, 0.114]
Slugging percentage	0.372	0.456	0.404	0.374	0.033	−0.082	0.115 [0.024, 0.206]
On base	0.336	0.387	0.370	0.353	0.034	−0.034	0.068 [0.024, 0.111]

This table summarizes the University of Cincinnati [Cin] performance against Big East [BE] teams and the Big East teams' performance parameters.

In addition, Table 4, we examined those games played only within the Big East conference, and again we saw an increase for Cincinnati in batting average from 2010 to 2011 of 0.045 versus the remainder of the Big East which got worse by 0.029, a difference of 0.074 [0.008, 0.139], p = 0.03. Slugging percentage; Cincinnati improved 0.009, the remainder of the Big East decreased 0.095, difference 0.104 [−0.019, 0.227], p = 0.09. On base percentage; Cincinnati improved 0.065, the rest of the Big East decreased 0.009, difference 0.074 [−0.007, 0.155], p = 0.07.

**Table 4 pone-0029109-t004:** Comparison of 2010 in conference season to 2011 in conference season: Cincinnati [Cin] versus the remainder of the Big East [BE].

	2010		2011		Change		Difference [95% CI]
	Cin	BE	Cin	BE	Cin	BE	
Batting average	0.233	0.294	0.278	0.265	0.045	−0.029	0.074 [0.008, 0.139]
Slugging percentage	0.342	0.440	0.351	0.345	0.009	−0.095	0.104 [−0.019, 0.227]
On base	0.316	0.371	0.381	0.362	0.065	−0.009	0.074 [−0.007, 0.155]

This table summarizes the University of Cincinnati [Cin] performance against Big East [BE] teams and the Big East teams' performance parameters.

Sensitivity analysis involved examining the changes from the 2009 to the 2011 season, which involves more changes in team members and larger anticipated differences in maturation. Again improvements for Cincinnati were seen compared to the remainder of the Big East conference teams, Table 5.

**Table 5:** Sensitivity analysis comparing 2009 in conference season to 2011 in conference season: Cincinnati [Cin] compared to the remainder of the Big East [BE].

	2009		2011		Change		Difference [95% CI]
	Cin	BE	Cin	BE	Cin	BE	
Batting average	.266	.303	.278	.265	.012	−.038	.050 [.016, −.004]
Slugging percentage	.432	.452	.351	.345	−.081	−.107	.026 [.135, −.083]
On base	.357	.380	.381	.362	.024	−.018	.042 [.128, −.045]

This table summarizes the University of Cincinnati performance against Big East teams and the Big East teams' performance parameters.

Some non-batting parameters that might have benefited from vision training also were observed. Errors decreased by 15% while fielding assists increased 8%. For the opponents the assists decreased by 8 % while errors were largely unchanged. University of Cincinnati errors was 90 and 76 while opponent errors was 54 and 57 in 2010 and 2011 respectively]. Thus some defensive parameters were possibly improved.

In the 2011 season the offense scored at least once every game resulting in a season with zero shutouts against the University of Cincinnati. This is the first shutoutless season the team had since joining the Big East and going back to before 1996.

### Limitations

Changes in batting average, slugging percentage and on base percentage could occur year to year for a variety of reasons. Players remaining on the team mature and improve, and new players may come from a stronger recruiting year. However, there is no reason to believe that this would change more positively for the University of Cincinnati than overall for the other teams within the Big East conference. Concurrent with the institution of this enhanced vision performance training the aluminum bats were changed in 2011 to behave more like wooden bats by the NCAA for all teams. So the bat change was uniform across all teams resulting in a decrement in batting average for all the other teams. Despite this change the batting statistics for the University of Cincinnati were significantly improved compared to the remainder of the Big East, however the potential confounding cannot be dismissed.

## Discussion

We believe that two critical parameters for any batter and their baseball team is batting average and on base percentage [10]. A player must be on base to score and hitting helps drive in runs. So we will examine those two parameters first. The University of Cincinnati baseball team had a substantial 0.034 point improvement in batting average from 2010 to 2011 where the opponents had a modest drop of 0.008. In tables 1 and 2 we present comparisons of the University of Cincinnati performance compared to our opponents in those games. In the tables 3 to [Table pone-0029109-t004] 5 we present data for the Big East teams. The opponents from year to year are largely the same teams and the two teams play under simultaneous conditions, so using the opponents performance parameters while playing against the University of Cincinnati as a control (given similar number of games and game conditions) is being used here.

The US Air Force Academy introduced a similar visual enhancement training program for their baseball team in 1994. The team batting average increased from 0.319 in 1993 to 0.360 in 1994 and they lead the nation in hitting. The team slugging percentage also increased from 0.487 to 0.623 as home runs increased from 32 to 76. These improvements were accomplished with 18 of 21 players returning from the 1993 season [Dr. Michael F. Zupan; personal communication and [11]. It thus appears that other teams when instituting a vision training regimen have seen similar improvement in batting parameters.

The improvement in the batting average is therefore striking because we assume that the main training difference is the addition of vision training [12]. Several of the vision training activities may be quite relevant to improved batting average. First, the Dynavision is a training tool for improving eye-hand coordination and the speed of that coordination [7]. Second, the near far drill assists in the ability of the eyes to focus from a distance; like the pitcher's hand to close in; the ball at the plate. The rotary training may also help the batting average because it provides a mechanism of training the eyes to track a moving object. Better tracking a moving object like the ball in the pitcher's hand or as it arcs to the plate will improve making contact with the ball.

Strobe training assists the brain in visualizing where the ball is going and the ball's rate. The brain is able to process the information the eye sends to it faster, and in that process when the Strobes training is discontinued, the ball's motion appears slower. This is an adaptation response of the brain and the duration with which the adaption remains is yet to be determined.

The slugging percentage for the University of Cincinnati improved with vision training. Strikingly there was a fall in the slugging percentage for the University's opponents. When one compares the batting averages for all the other Big East Teams all the teams had a fall in the team batting average from 2010 to 2011. The University of Cincinnati was the only team of the 12 Big East teams to show any improvement in batting average and that increase was 0.034.

For the 2011 season the NCAA instituted a new standard for bats to make the aluminum bats behave more like wood bats. This change appeared to adversely affect the batting averages of teams in the Big East, whereas there was an improvement in most batting parameters for the University of Cincinnati. Interestingly in the year the new bats were initiated the University of Cincinnati baseball team recorded its first shutoutless season in 15 years. Consistent scoring by the offense is, we believe, indicative of a strong batting foundation for the team's batters.

Many experienced vision training practitioners will recognize that a host of additional training modalities could be employed. Things like I-span, ball numbering, and others can be added to a training regimen. We chose our drills for training batters based on the skills needed by the batters, the time needed/allotted for training and the resources available. Our choices of vision training modalities were also, in part, based on discussions with Dr. Zupan and Al Wile of the Air Force Acadamy.

Future work with batters and hitting with vision training could investigate the dose response for vision training benefits as well as evaluation of other drills that could be used [12]. At this time our philosophy is that vision training for improved batting performance can be done in many ways. What is reported here is simply a reflection of the apparent effectiveness of the vision training employed at the University of Cincinnati. Further, coaches and athletic trainers will be able to make adjustments to the program used by the University of Cincinnati to be more intense should players need it or shorter in duration as schedules dictate.

The training was designed to improve various ocular motor parameters. The muscles in the eyes can be trained and conditioned to perform better and faster in focusing and tracking objects such as baseballs. During the preseason training sessions it was common for athletes to report delayed onset muscle soreness in the eyes. This sensation was transient and is consistent with muscle conditioning that diminished with training as the season progressed.

Baseball batters have about 0.17 seconds to decide to hit a pitch and choose where to swing etc. The time it takes the pitch to cross the plate is about 0.4 seconds. This is a complicated spatial and geometric decision on a tight time frame [13]. The average batter can expect to have only 1 or 2 pitches that are truly hittable in any at bat. So choosing that pitch is paramount for their success. Spotting the pitchers hand position on the ball, the velocity and type of pitch needs to be achieved rapidly and with great fidelity. “I can still remember my Dad saying, watch the ball pass the Pitcher ear” [Johnny Bench; Personal communication]. Watching the ball before the pitcher's release can buy time and information for the batter [13]. This is an important snapshot of information from the pitcher available to the hitter and with vision training the benefit of that information can be improved and better utilized.

How does so much vision and processing happen within that short 0.4 second time frame? The eyes account for 80% of the information taken into the brain. Having good vision and training that vision to be proficient may provide the batter with a competitive edge [6], [8]. It is estimated that the average visual acuity of a professional baseball player is 20/12 whereas college players are of the order of 20/15 [6]. Wade Boggs talked about having 20/10 vision [http://www.nytimes.com/1996/04/21/sports/baseball-puckett-facing-life-outside-baseball-keeps-fighting.html?pagewanted=4]. He went on to imply that as his vision degraded with age to 20/20 he wanted it corrected back to 20/10. This reinforces the importance of good and proficient vision for performance [14]. We know that 20/20 is “normal” or what physicians tend to strive for with vision correction. Therefore most people who wear glasses are corrected to the 20/20 range. Factors can play a part in hitting as well such as background, lighting and audible distractions. The idea that vision can be improved to give you that millisecond advantage is what can be attained through vision correction and vision training, with the latter presented in the current manuscript.

We posit that the vision training program makes the batter able to spot ball and pitcher's finger position and thereby spot pitches better and earlier. The Tachistoscope training may help with the “snapshot” of information from the pitcher holding the ball and the rotary may help the batter follow the ball, but we firmly believe it is the synthesis of the training program that improved the whole team's batting abilities.

If you do not start with the basics of batting, there will be a lot of swinging and misses or foul balls, this is a form of vision training by trial and error. Joe Schultz in 1969 was talking about how hard batting was when he was quoted as saying, "Well, boys, it's a round ball and a round bat and you got to hit the ball square." It might seem simple to have a ball thrown to you in a lob and make effective contact on the swing. The real challenges begin when velocity is increased from various angles and then have movement of the ball brought into the equation. Movement of the ball in this case is changing the trajectory away from a traditional parabola; generally caused by spin and friction from the ball's laces during spin [3]. Speeds change from pitch to pitch and within a few milliseconds you must identify; spin, speed and trajectory and then have the cognitive training go into action by sending appropriate signals to the nerves and muscles to perform a proficient swing.

We have trained athletes and personally tried these programs and are very aware of their benefits; along with the needs or deficiency of certain athletes' vision performance. The anticipated benefits of vision training are not exclusive to Baseball [1]. We believe that Football players will be able to improve their reaction times, but this speculation will need to be validated. There are few sports that could not benefit from some form of vision training.

Concerning non-batting parameters the apparent increase in assists [as well as decreased errors] is something that may partially be attributable to improved visual motor coordination. An assist often requires a rapid and cohesive exchange of tracking ball into mitt and ball into hand for an effective throw. Improved eye-hand coordination may decrease fielding errors and dropped balls but also may help with assists as the action on the field may appear slower and easier to follow for the vision trained athlete who can then make plays better. It may be that with vision training the presentation of the ball into the mitt may be more proficient such that the exchange to the throw can be faster with fewer mistakes resulting in fewer recorded errors and increased assists. One may also speculate that these defensive parameters may be benefiting somewhat by the vision training because of improved self confidence as well as improved eye-hand coordination. This post hoc analysis is not conclusive, but suggests added benefits from vision training.

The University of Cincinnati baseball team, coaches and vision performance team have concluded that our vision training program had positive benefits in the offensive game including batting and may be providing improved play on defense as well. Vision training is becoming part of our pre-season and in season conditioning program as well as for warmups.
